# Supplementary material for: Rapid Phenotype-Driven Gene Sequencing with the NeoSeq Panel: A Diagnostic Tool for Critically Ill Newborns with Suspected Genetic Disease
Source: J Clin Med. 2020 Jul 23;9(8):2362. doi: 10.3390/jcm9082362 (PMC7464859; doi:10.3390/jcm9082362)
Supplement: Supplementary file 1 [file jcm-09-02362-s001.zip › Figure S1.pptx]

## Slide 1
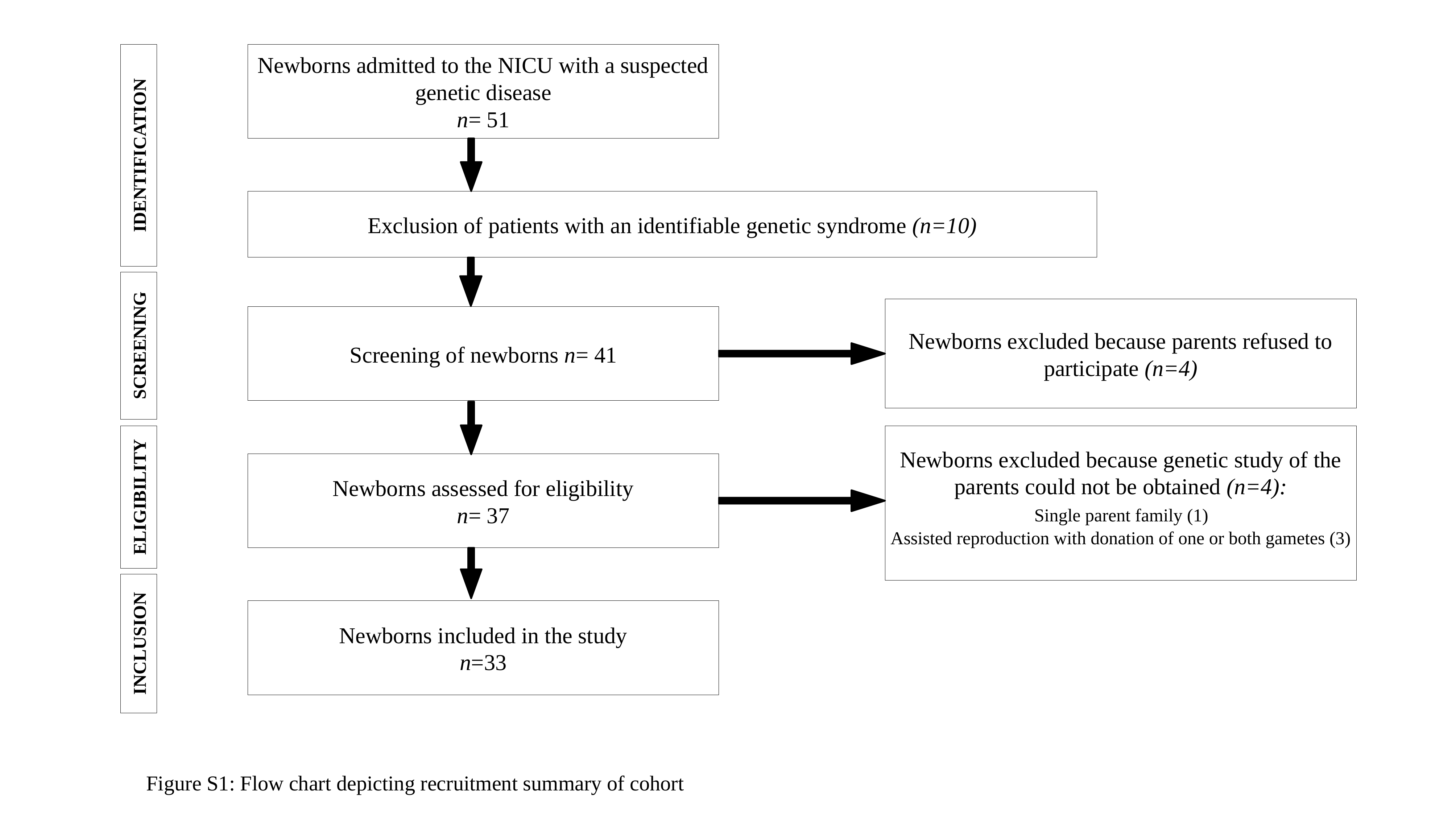

Newborns admitted to the NICU with a suspected genetic disease
n= 51
IDENTIFICATION
Exclusion of patients with an identifiable genetic syndrome (n=10)
Newborns excluded because parents refused to participate (n=4)
Screening of newborns n= 41
SCREENING
Newborns excluded because genetic study of the parents could not be obtained (n=4):
 Single parent family (1)
Assisted reproduction with donation of one or both gametes (3)
Newborns assessed for eligibility
n= 37
ELIGIBILITY
Newborns included in the study
n=33
INCLUSION
Figure S1: Flow chart depicting recruitment summary of cohort
