# Supplementary material for: Rapid Phenotype-Driven Gene Sequencing with the NeoSeq Panel: A Diagnostic Tool for Critically Ill Newborns with Suspected Genetic Disease
Source: J Clin Med. 2020 Jul 23;9(8):2362. doi: 10.3390/jcm9082362 (PMC7464859; doi:10.3390/jcm9082362)
Supplement: Supplementary file 1 [file jcm-09-02362-s001.zip › Table S1.docx]

Table S1 – List of genes included in NeoSeq with the corresponding OMIM entry.

| AARS1 | *601065 | ACOX1 | *609751 | AGA | *613228 | ALDH6A1 | *603178 | ANO10 | *613726 | ARID1A | *603024 |
| --- | --- | --- | --- | --- | --- | --- | --- | --- | --- | --- | --- |
| AARS2 | *612035 | ACSF3 | *614245 | AGK | *610345 | ALDH7A1 | *107323 | ANO3 | *610110 | ARID1B | *614556 |
| AASS | *605113 | ACSL4 | *300157 | AGL | *610860 | ALDOA | *103850 | ANO5 | *608662 | ARL13B | *608922 |
| ABAT | *137150 | ACTA1 | *102610 | AGMO | *613738 | ALDOB | *612724 | AP1S2 | *300629 | ARL3 | *604695 |
| ABCB11 | *603201 | ACTB | *102630 | AGO1 | *606228 | ALG1 | *605907 | AP3B2 | *602166 | ARL6 | *608845 |
| ABCB7 | *300135 | ACTG1 | *102560 | AGPS | *603051 | ALG11 | *613666 | AP4B1 | *607245 | ARL6IP1 | *607669 |
| ABCC8 | *600509 | ADA | *608958 | AGRN | *103320 | ALG12 | *607144 | AP4E1 | *607244 | ARMC4 | *615408 |
| ABCD1 | *300371 | ADAM22 | *603709 | AGXT | *604285 | ALG13 | *300776 | AP4M1 | *602296 | ARMC9 | *617612 |
| ABCD3 | *170995 | ADAMTS2 | *604539 | AGXT2 | *612471 | ALG14 | *612866 | AP4S1 | *607243 | ARSA | *607574 |
| ABCD4 | *603214 | ADAR | *146920 | AHCY | *180960 | ALG2 | *607905 | AP5Z1 | *613653 | ARSB | *611542 |
| ABHD12 | *613599 | ADAT3 | *615302 | AHDC1 | *615790 | ALG3 | *608750 | APBB1 | *602709 | ARSI | *610009 |
| ABHD5 | *604780 | ADCK3 | *606980 | AHI1 | *608894 | ALG6 | *604566 | APOA1BP | *608862 | ARV1 | *611647 |
| ACAA1 | *604054 | ADCY1 | *103072 | AIFM1 | *300169 | ALG8 | *608103 | APOL2 | *607252 | ARX | *300382 |
| ACAD8 | *604773 | ADCY5 | *600293 | AIMP1 | *603605 | ALG9 | *606941 | APOL4 | *607254 | ASAH1 | *613468 |
| ACAD9 | *611103 | ADCY6 | *600294 | AIMP2 | *600859 | ALMS1 | *606844 | APOPT1 | *616003 | ASCC1 | *614215 |
| ACADM | *607008 | ADD3 | *601568 | AKR1B15 | *616336 | ALPK3 | *617608 | APPL1 | *604299 | ASH1L | *607999 |
| ACADS | *606885 | ADGRG1 | *604110 | AKR1D1 | *604741 | ALS2 | *606352 | APRT | *102600 | ASL | *608310 |
| ACADSB | *600301 | ADGRG6 | *612243 | AKT1 | *164730 | ALX4 | *605420 | APTX | *606350 | ASNS | *108370 |
| ACADVL | *609575 | ADGRV1 | *602851 | AKT2 | *164731 | AMACR | *604489 | ARCN1 | *600820 | ASPA | *608034 |
| ACAT1 | *607809 | ADK | *102750 | AKT3 | *611223 | AMPD1 | *102770 | ARFGEF2 | *605371 | ASPM | *605481 |
| ACAT2 | *100678 | ADNP | *611386 | ALAD | *125270 | AMPD2 | *102771 | ARG1 | *608313 | ASS1 | *603470 |
| ACBD5 | *616618 | ADSL | *608222 | ALAS2 | *301300 | AMT | *238310 | ARHGEF10 | *608136 | ATAD1 | *614452 |
| ACER3 | *617036 | ADSSL1 | *612498 | ALDH18A1 | *138250 | ANK3 | *600465 | ARHGEF15 | *608504 | ATAD3A | *612316 |
| ACHE | *100740 | AEBP1 | *602981 | ALDH3A2 | *609523 | ANKH | *605145 | ARHGEF2 | *607560 | ATAD3B | *612317 |
| ACMSD | *608889 | AFF2 | *300806 | ALDH4A1 | *606811 | ANKLE2 | *616062 | ARHGEF6 | *300267 | ATAD3C | *617227 |
| ACO2 | *100850 | AFG3L2 | *604581 | ALDH5A1 | *610045 | ANKRD11 | *611192 | ARHGEF9 | *300429 | ATCAY | *608179 |
| ATG5 | *604261 | ATPAF1 | *608917 | BCKDK | *614901 | CACNA1C | *114205 | CCDC88A | *609736 | CHAMP1 | *616327 |
| ATL1 | *606439 | ATPAF2 | *608918 | BCL11A | *606557 | CACNA1D | *114206 | CCDC88C | *611204 | CHAT | *118490 |
| ATL3 | *609369 | ATR | *601215 | BCL11B | *606558 | CACNA1G | *604065 | CCND2 | *123833 | CHCHD10 | *615903 |
| ATM | *607585 | ATRN | *603130 | BCS1L | *603647 | CACNA1H | *607904 | CCT5 | *610150 | CHD1 | *602118 |
| ATP13A2 | *610513 | ATRX | *300032 | BDP1 | *607012 | CACNA1S | *114208 | CD320 | *606475 | CHD2 | *602119 |
| ATP1A2 | *182340 | ATXN2 | *601517 | BEAN1 | *612051 | CACNA2D1 | *114204 | CDH15 | *114019 | CHD7 | *608892 |
| ATP1A3 | *182350 | AUH | *600529 | BICD2 | *609797 | CACNA2D2 | *607082 | CDH23 | *605516 | CHD8 | *610528 |
| ATP1B1 | *182330 | AUTS2 | *607270 | BIN1 | *601248 | CACNB4 | *601949 | CDK13 | *603309 | CHI3L1 | *601525 |
| ATP2A1 | *108730 | B3GALNT2 | *610194 | BLK | *191305 | CACNG2 | *602911 | CDK5 | *123831 | CHKB | *612395 |
| ATP2B3 | *300014 | B4GALNT1 | *601873 | BMP1 | *112264 | CAD | *114010 | CDK5RAP2 | *608201 | CHMP1A | *164010 |
| ATP5E | *606153 | B4GALT1 | *137060 | BOLA3 | *613183 | CAMK2A | *114078 | CDK6 | *603368 | CHMP1B | *606486 |
| ATP5F1A | *164360 | B4GALT7 | *604327 | BPTF | *601819 | CAMK2B | *607707 | CDKL5 | *300203 | CHRNA1 | *100690 |
| ATP5F1B | *102910 | B4GAT1 | *605517 | BRAF | *164757 | CAMTA1 | *611501 | CDON | *608707 | CHRNA2 | *118502 |
| ATP5F1C | *108729 | B9D1 | *614144 | BRAT1 | *614506 | CAPN1 | *114220 | CEL | *114840 | CHRNA4 | *118504 |
| ATP5F1D | *603150 | B9D2 | *611951 | BRF1 | *604902 | CAPN3 | *114240 | CENPE | *117143 | CHRNB1 | *100710 |
| ATP5G1 | *603192 | BAG3 | *603883 | BRPF1 | *602410 | CARS2 | *612800 | CENPF | *600236 | CHRNB2 | *118507 |
| ATP5G2 | *603193 | BBIP1 | *613605 | BRWD3 | *300553 | CASK | *300172 | CENPJ | *609279 | CHRND | *100720 |
| ATP5G3 | *602736 | BBS1 | *209901 | BSCL2 | *606158 | CASQ1 | *114250 | CEP104 | *616690 | CHRNE | *100725 |
| ATP5PB | *603270 | BBS10 | *610148 | BSND | *606412 | CASR | *601199 | CEP120 | *613446 | CHRNG | *100730 |
| ATP5PO | *600828 | BBS12 | *610683 | BTD | *609019 | CAV3 | *601253 | CEP135 | *611423 | CHST14 | *608429 |
| ATP6AP2 | *300556 | BBS2 | *606151 | BVES | *604577 | CBL | *165360 | CEP152 | *613529 | CIB2 | *605564 |
| ATP6V0A2 | *611716 | BBS4 | *600374 | C12ORF65 | *613541 | CBS | *613381 | CEP164 | *614848 | CIC | *612082 |
| ATP6V0A4 | *605239 | BBS5 | *603650 | C19ORF12 | *614297 | CC2D1A | *610055 | CEP290 | *610142 | CISD2 | *611507 |
| ATP6V1A | *607027 | BBS7 | *607590 | C8ORF37 | *614477 | CC2D2A | *612013 | CEP41 | *610523 | CIT | *605629 |
| ATP6V1B1 | *192132 | BBS9 | *607968 | CA5A | *114761 | CCDC115 | *613734 | CEP63 | *614724 | CIZ1 | *611420 |
| ATP7A | *300011 | BCAP31 | *300398 | CA8 | *114815 | CCDC174 | *616735 | CEP89 | *615470 | CLCN1 | *118425 |
| ATP7B | *606882 | BCKDHA | *608348 | CACNA1A | *601011 | CCDC28B | *610162 | CERS1 | *606919 | CLCN2 | *600570 |
| ATP8A2 | *605870 | BCKDHB | *248611 | CACNA1B | *601012 | CCDC78 | *614666 | CFL2 | *601443 | CLCN4 | *302910 |
| CLCN6 | *602726 | COG5 | *606821 | COL9A2 | *120260 | COX8A | *123870 | CTSD | *116840 | DDC | *107930 |
| CLIC2 | *300138 | COG6 | *606977 | COLEC11 | *612502 | CP | *117700 | CTSF | *603539 | DDHD1 | *614603 |
| CLN3 | *607042 | COG7 | *606978 | COLQ | *603033 | CPA6 | *609562 | CTTNBP2 | *609772 | DDHD2 | *615003 |
| CLN5 | *608102 | COG8 | *606979 | COMT | +116790 | CPLANE1 | *614571 | CTU2 | *617057 | DDOST | *602202 |
| CLN6 | *606725 | COL11A1 | *120280 | COQ2 | *609825 | CPLX1 | *605032 | CUL4B | *300304 | DDX3X | *300160 |
| CLN8 | *607837 | COL11A2 | *120290 | COQ3 | *605196 | CPOX | *612732 | CUX2 | *610648 | DEAF1 | *602635 |
| CLP1 | *608757 | COL12A1 | *120320 | COQ4 | *612898 | CPS1 | *608307 | CWF19L1 | *616120 | DENND5A | *617278 |
| CLPB | *616254 | COL13A1 | *120350 | COQ5 | *616359 | CPT1A | *600528 | CXORF56 | *301012 | DEPDC5 | *614191 |
| CLPP | *601119 | COL18A1 | *120328 | COQ6 | *614647 | CPT1C | *608846 | CYC1 | *123980 | DES | *125660 |
| CLPX | *615611 | COL1A1 | +120150 | COQ7 | *601683 | CPT2 | *600650 | CYCS | *123970 | DGAT2 | *606983 |
| CLRN1 | *606397 | COL1A2 | *120160 | COQ8B | *615567 | CRADD | *603454 | CYFIP2 | *606323 | DGUOK | *601465 |
| CLTC | *118955 | COL2A1 | +120140 | COQ9 | *612837 | CRAT | *600184 | CYP26B1 | *605207 | DHCR24 | *606418 |
| CNKSR2 | *300724 | COL3A1 | *120180 | COX10 | *602125 | CRBN | *609262 | CYP27A1 | *606530 | DHCR7 | *602858 |
| CNNM2 | *607803 | COL4A1 | *120130 | COX11 | *603648 | CRTAP | *605497 | CYP2U1 | *610670 | DHDDS | *608172 |
| CNOT3 | *604910 | COL4A2 | *120090 | COX14 | *614478 | CRYAB | *123590 | CYP7B1 | *603711 | DHFR | *126060 |
| CNPY3 | *610774 | COL4A3 | *120070 | COX15 | *603646 | CSDE1 | *191510 | D2HGDH | *609186 | DHTKD1 | *614984 |
| CNTN1 | *600016 | COL4A3BP | *604677 | COX16 | *618064 | CSF1R | *164770 | DAB1 | *603448 | DHX30 | *616423 |
| CNTN2 | *190197 | COL4A4 | *120131 | COX17 | *604813 | CSNK1G1 | *606274 | DAG1 | *128239 | DIAPH1 | *602121 |
| CNTNAP1 | *602346 | COL4A5 | *303630 | COX18 | *610428 | CSNK2B | *115441 | DAO | *124050 | DISC1 | *605210 |
| CNTNAP2 | *604569 | COL4A6 | *303631 | COX19 | *610429 | CSPP1 | *611654 | DAOA | *607408 | DISP1 | *607502 |
| COA5 | *613920 | COL5A1 | *120215 | COX20 | *614698 | CSTB | *601145 | DARS1 | *603084 | DLAT | *608770 |
| COA6 | *614772 | COL5A2 | *120190 | COX4I1 | *123864 | CTBP1 | *602618 | DARS2 | *610956 | DLD | *238331 |
| COA7 | *615623 | COL5A3 | *120216 | COX4I2 | *607976 | CTC1 | *613129 | DBT | *248610 | DLG3 | *300189 |
| COASY | *609855 | COL6A1 | *120220 | COX6A1 | *602072 | CTCF | *604167 | DCAF17 | *612515 | DLGAP4 | *616191 |
| COCH | *603196 | COL6A2 | *120240 | COX6A2 | *602009 | CTH | *607657 | DCAF8 | *615820 | DLL1 | *606582 |
| COG1 | *606973 | COL6A3 | *120250 | COX6B1 | *124089 | CTNNA2 | *114025 | DCC | *120470 | DMD | *300377 |
| COG2 | *606974 | COL6A6 | *616613 | COX6B2 | *618127 | CTNNB1 | *116806 | DCHS1 | *603057 | DNA2 | *601810 |
| COG4 | *606976 | COL9A1 | *120210 | COX7B | *300885 | CTSA | *613111 | DCX | *300121 | DNAJB2 | *604139 |
| DNAJB6 | *611332 | DSTYK | *612666 | ELAC2 | *605367 | EXOSC9 | *606180 | FGF8 | *600483 | FRMPD4 | *300838 |
| DNAJC12 | *606060 | DYM | *607461 | ELOVL4 | *605512 | EXT2 | *608210 | FGFR1 | *136350 | FRRS1L | *604574 |
| DNAJC19 | *608977 | DYNC1H1 | *600112 | ELOVL5 | *611805 | EYA1 | *601653 | FGFR2 | *176943 | FTCD | *606806 |
| DNAJC3 | *601184 | DYRK1A | *600855 | ELP1 | *603722 | EYA4 | *603550 | FGFR3 | *134934 | FTL | *134790 |
| DNAJC5 | *611203 | DYSF | *603009 | ELP2 | *616054 | EZH2 | *601573 | FH | *136850 | FTSJ1 | *300499 |
| DNM1 | *602377 | E4F1 | *603022 | EMD | *300384 | FA2H | *611026 | FHL1 | *300163 | FUCA1 | *612280 |
| DNM1L | *603850 | EARS2 | *612799 | EMILIN1 | *130660 | FAH | *613871 | FIG4 | *609390 | FUS | *137070 |
| DNM2 | *602378 | EBF3 | *607407 | EML1 | *602033 | FAM111B | *615584 | FKBP10 | *607063 | FUT8 | *602589 |
| DNMT1 | *126375 | EBP | *300205 | EMX2 | *600035 | FAM126A | *610531 | FKBP14 | *614505 | G6PC | *613742 |
| DNMT3A | *602769 | ECEL1 | *605896 | ENTPD1 | *601752 | FARS2 | *611592 | FKRP | *606596 | GAA | *606800 |
| DOCK3 | *603123 | ECHS1 | *602292 | EOMES | *604615 | FARSB | *609690 | FKTN | *607440 | GABBR2 | *607340 |
| DOCK7 | *615730 | EDC3 | *609842 | EPB41L1 | *602879 | FAS | *134637 | FLAD1 | *610595 | GABRA1 | *137160 |
| DOCK8 | *611432 | EDN3 | *131242 | EPM2A | *607566 | FASTKD2 | *612322 | FLNA | *300017 | GABRA5 | *137142 |
| DOK7 | *610285 | EDNRB | *131244 | EPRS1 | *138295 | FAT2 | *604269 | FLNC | *102565 | GABRB1 | *137190 |
| DOLK | *610746 | EEF1A2 | *602959 | ERAL1 | *607435 | FAT4 | *612411 | FLVCR1 | *609144 | GABRB2 | *600232 |
| DPAGT1 | *191350 | EEF2 | *130610 | ERBB3 | *190151 | FBN1 | *134797 | FMN2 | *606373 | GABRB3 | *137192 |
| DPM1 | *603503 | EFHC1 | *608815 | ERBB4 | *600543 | FBN2 | *612570 | FMO3 | *136132 | GABRD | *137163 |
| DPM2 | *603564 | EFNB1 | *300035 | ERF | *611888 | FBP1 | *611570 | FMR1 | *309550 | GABRG2 | *137164 |
| DPM3 | *605951 | EFTUD2 | *603892 | ERGIC1 | *617946 | FBXL4 | *605654 | FOLR1 | *136430 | GAD1 | *605363 |
| DPP6 | *126141 | EGR2 | *129010 | ERLIN1 | *611604 | FBXO11 | *607871 | FOXA2 | *600288 | GALC | *606890 |
| DPYD | *612779 | EIF2AK2 | *176871 | ERLIN2 | *611605 | FBXO31 | *609102 | FOXG1 | *164874 | GALE | *606953 |
| DPYS | *613326 | EIF2B1 | *606686 | ERMARD | *615532 | FDFT1 | *184420 | FOXH1 | *603621 | GALK1 | *604313 |
| DRD3 | *126451 | EIF2B2 | *606454 | ETFA | *608053 | FDX2 | *614585 | FOXI1 | *601093 | GALNS | *612222 |
| DRD4 | *126452 | EIF2B3 | *606273 | ETFB | *130410 | FECH | *612386 | FOXP1 | *605515 | GALT | *606999 |
| DRD5 | *126453 | EIF2B4 | *606687 | ETFDH | *231675 | FGD1 | *300546 | FOXP2 | *605317 | GAN | *605379 |
| DRP2 | *300052 | EIF2B5 | *603945 | ETHE1 | *608451 | FGD4 | *611104 | FOXRED1 | *613622 | GARS1 | *600287 |
| DSCAM | *602523 | EIF2S3 | *300161 | EVL | *616912 | FGF12 | *601513 | FREM1 | *608944 | GAS1 | *139185 |
| DST | *113810 | EIF4E | *133440 | EXOSC3 | *606489 | FGF14 | *601515 | FRMD4A | *616305 | GATAD2B | *614998 |
| GBA | *606463 | GLI3 | *165240 | GPC3 | *300037 | GYS2 | *138571 | HIKESHI | *614908 | HSPB1 | *602195 |
| GBA2 | *609471 | GLIS2 | *608539 | GPHN | *603930 | GZF1 | *613842 | HINT1 | *601314 | HSPB3 | *604624 |
| GBE1 | *607839 | GLIS3 | *610192 | GPR88 | *607468 | HACD1 | *610467 | HINT3 | *609998 | HSPB8 | *608014 |
| GCDH | *608801 | GLRA1 | *138491 | GPRASP1 | *300417 | HACE1 | *610876 | HIVEP2 | *143054 | HSPD1 | *118190 |
| GCH1 | *600225 | GLRB | *138492 | GPSM2 | *609245 | HADH | *601609 | HK1 | *142600 | HSPE1 | *600141 |
| GCK | *138079 | GLRX5 | *609588 | GPT2 | *138210 | HADHA | *600890 | HLCS | *609018 | HSPG2 | *142461 |
| GCSH | *238330 | GLUD1 | *138130 | GRB14 | *601524 | HADHB | *143450 | HMBS | *609806 | HTR2A | *182135 |
| GDAP1 | *606598 | GLUL | *138290 | GRHL2 | *608576 | HAL | *609457 | HMGCL | *613898 | HTRA1 | *602194 |
| GDF6 | *601147 | GLYCTK | *610516 | GRHPR | *604296 | HARS1 | *142810 | HMGCS2 | *600234 | HTRA2 | *606441 |
| GDI1 | *300104 | GM2A | *613109 | GRIA3 | *305915 | HARS2 | *600783 | HNF1A | *142410 | HUWE1 | *300697 |
| GEMIN4 | *606969 | GMPPA | *615495 | GRIA4 | *138246 | HBA1 | *141800 | HNF1B | *189907 | HYAL1 | *607071 |
| GFAP | *137780 | GMPPB | *615320 | GRID2 | *602368 | HCCS | *300056 | HNF4A | *600281 | IARS1 | *600709 |
| GFER | *600924 | GNAL | *139312 | GRIK2 | *138244 | HCFC1 | *300019 | HNMT | *605238 | IARS2 | *612801 |
| GFM1 | *606639 | GNAO1 | *139311 | GRIN1 | *138249 | HCN1 | *602780 | HNRNPA1 | *164017 | IBA57 | *615316 |
| GFM2 | *606544 | GNAQ | *600998 | GRIN2A | *138253 | HCN2 | *602781 | HNRNPA2B1 | *600124 | ICK | *612325 |
| GFPT1 | *138292 | GNB1 | *139380 | GRIN2B | *138252 | HCN4 | *605206 | HNRNPDL | *607137 | IDH2 | *147650 |
| GGT1 | *612346 | GNB4 | *610863 | GRIN2D | *602717 | HDAC8 | *300269 | HNRNPH1 | *601035 | IDS | *300823 |
| GIPC3 | *608792 | GNB5 | *604447 | GRM1 | *604473 | HECW2 | *617245 | HNRNPH2 | *300610 | IDUA | *252800 |
| GJB1 | *304040 | GNE | *603824 | GRM5 | *604102 | HEPACAM | *611642 | HNRNPU | *602869 | IER3IP1 | *609382 |
| GJB2 | *121011 | GNMT | *606628 | GRN | *138945 | HERC2 | *605837 | HOGA1 | *613597 | IFIH1 | *606951 |
| GJB6 | *604418 | GNPAT | *602744 | GSDME | *608798 | HESX1 | *601802 | HOXA1 | *142955 | IFITM5 | *614757 |
| GJC2 | *608803 | GNPTAB | *607840 | GSTZ1 | *603758 | HEXA | *606869 | HPCA | *142622 | IFT122 | *606045 |
| GLA | *300644 | GNPTG | *607838 | GTPBP3 | *608536 | HEXB | *606873 | HPD | *609695 | IFT27 | *615870 |
| GLB1 | *611458 | GNS | *607664 | GUF1 | *617064 | HFE | *613609 | HPRT1 | *308000 | IFT74 | *608040 |
| GLDC | *238300 | GOLGA2 | *602580 | GUSB | *611499 | HGD | *607474 | HRAS | *190020 | IGBP1 | *300139 |
| GLDN | *608603 | GOSR2 | *604027 | GYG1 | *603942 | HGF | *142409 | HSD17B10 | *300256 | IGHMBP2 | *600502 |
| GLE1 | *603371 | GOT2 | *138150 | GYG2 | *300198 | HGSNAT | *610453 | HSD17B4 | *601860 | IL11RA | *600939 |
| GLI2 | *165230 | GPAA1 | *603048 | GYS1 | *138570 | HIBCH | *610690 | HSD3B7 | *607764 | IL1RAPL1 | *300206 |
| IMPA1 | *602064 | KCNA2 | *176262 | KIF11 | *148760 | LAMB2 | *150325 | LRP4 | *604270 | MCCC2 | *609014 |
| INAVA | *618051 | KCNB1 | *600397 | KIF14 | *611279 | LAMC3 | *604349 | LRPPRC | *607544 | MCEE | *608419 |
| INF2 | *610982 | KCNC1 | *176258 | KIF1A | *601255 | LAMP2 | *309060 | LRSAM1 | *610933 | MCM3AP | *603294 |
| INPP5E | *613037 | KCNC3 | *176264 | KIF1B | *605995 | LARGE1 | *603590 | LTC4S | *246530 | MCOLN1 | *605248 |
| INPP5K | *607875 | KCND3 | *605411 | KIF1C | *603060 | LARS2 | *604544 | LYRM4 | *613311 | MCPH1 | *607117 |
| INS | *176730 | KCNE1 | *176261 | KIF22 | *603213 | LAS1L | *300964 | LYRM7 | *615831 | MDH1 | *154200 |
| INSR | *147670 | KCNH1 | *603305 | KIF2A | *602591 | LDB3 | *605906 | LZTFL1 | *606568 | MDH2 | *154100 |
| INVS | *243305 | KCNH5 | *605716 | KIF4A | *300521 | LDHA | *150000 | LZTR1 | *600574 | MECP2 | *300005 |
| IQCB1 | *609237 | KCNJ10 | *602208 | KIF5A | *602821 | LETM1 | *604407 | MAFA | *610303 | MECR | *608205 |
| IQSEC1 | *610166 | KCNJ11 | *600937 | KIF5C | *604593 | LGI1 | *604619 | MAG | *159460 | MED12 | *300188 |
| IQSEC2 | *300522 | KCNMA1 | *600150 | KIF7 | *611254 | LGI4 | *608303 | MAGEL2 | *605283 | MED13L | *608771 |
| IRF2BPL | *611720 | KCNQ1 | *607542 | KIRREL3 | *607761 | LIAS | *607031 | MAGI2 | *606382 | MED17 | *603810 |
| ISCA1 | *611006 | KCNQ2 | *602235 | KLC2 | *611729 | LIMS2 | *607908 | MAN1B1 | *604346 | MED20 | *612915 |
| ISCA2 | *615317 | KCNQ3 | *602232 | KLF11 | *603301 | LINGO1 | *609791 | MAN2B1 | *609458 | MED23 | *605042 |
| ISCU | *611911 | KCNQ4 | *603537 | KLHL15 | *300980 | LINS1 | *610350 | MANBA | *609489 | MED25 | *610197 |
| ISPD | *614631 | KCNQ5 | *607357 | KLHL40 | *615340 | LIPA | *613497 | MAOA | *309850 | MEF2C | *600662 |
| ITGA5 | *135620 | KCNT1 | *608167 | KLHL41 | *607701 | LIPT1 | *610284 | MAP2 | *157130 | MEGF10 | *612453 |
| ITGA7 | *600536 | KCNT2 | *610044 | KMT2B | *606834 | LIPT2 | *617659 | MAPK10 | *602897 | MEIS2 | *601740 |
| ITPA | *147520 | KCTD17 | *616386 | KMT5B | *610881 | LITAF | *603795 | MAPT | *157140 | MET | *164860 |
| ITPR1 | *147265 | KCTD7 | *611725 | KNL1 | *609173 | LMAN2L | *609552 | MARS1 | *156560 | METTL23 | *615262 |
| IVD | *607036 | KDM5B | *605393 | KPTN | *615620 | LMBRD1 | *612625 | MARS2 | *609728 | MFF | *614785 |
| JPH1 | *605266 | KDM5C | *314690 | KRAS | *190070 | LMNA | *150330 | MASP1 | *600521 | MFN1 | *608506 |
| KANK1 | *607704 | KDM6A | *300128 | KRIT1 | *604214 | LMNB1 | *150340 | MAT1A | *610550 | MFN2 | *608507 |
| KARS1 | *601421 | KIAA0556 | *616650 | KY | *605739 | LMNB2 | *150341 | MAT2A | *601468 | MFSD2A | *614397 |
| KAT6A | *601408 | KIAA0586 | *610178 | L1CAM | *308840 | LMOD3 | *616112 | MATR3 | *164015 | MFSD8 | *611124 |
| KATNB1 | *602703 | KIAA1109 | *611565 | L2HGDH | *609584 | LNPK | *610236 | MBD5 | *611472 | MGAT2 | *602616 |
| KBTBD13 | *613727 | KIAA1279 | *609367 | LAMA2 | *156225 | LONP1 | *605490 | MBOAT7 | *606048 | MGME1 | *615076 |
| KCNA1 | *176260 | KIDINS220 | *615759 | LAMB1 | *150240 | LRP2 | *600073 | MCCC1 | *609010 | MICU1 | *605084 |
| MID2 | *300204 | MRPL44 | *611849 | MYH14 | *608568 | NCAPH | *602332 | NDUFB5 | *603841 | NFIA | *600727 |
| MIPEP | *602241 | MRPS16 | *609204 | MYH2 | *160740 | NCKAP1 | *604891 | NDUFB6 | *603322 | NFS1 | *603485 |
| MIR17HG | *609415 | MRPS22 | *605810 | MYH3 | *160720 | NDE1 | *609449 | NDUFB7 | *603842 | NFU1 | *608100 |
| MITF | *156845 | MRPS23 | *611985 | MYH7 | *160760 | NDP | *300658 | NDUFB8 | *602140 | NGLY1 | *610661 |
| MKKS | *604896 | MRPS34 | *611994 | MYH8 | *160741 | NDRG1 | *605262 | NDUFB9 | *601445 | NHEJ1 | *611290 |
| MKS1 | *609883 | MRPS7 | *611974 | MYH9 | *160775 | NDST1 | *600853 | NDUFS1 | *157655 | NHLRC1 | *608072 |
| MLC1 | *605908 | MSMO1 | *607545 | MYO15A | *602666 | NDUFA1 | *300078 | NDUFS2 | *602985 | NIN | *608684 |
| MLYCD | *606761 | MSRB3 | *613719 | MYO18B | *607295 | NDUFA10 | *603835 | NDUFS3 | *603846 | NIPA1 | *608145 |
| MMAA | *607481 | MSTO1 | *617619 | MYO3A | *606808 | NDUFA11 | *612638 | NDUFS4 | *602694 | NIPA2 | *608146 |
| MMAB | *607568 | MSX2 | *123101 | MYO6 | *600970 | NDUFA12 | *614530 | NDUFS5 | *603847 | NIPBL | *608667 |
| MMACHC | *609831 | MTCH1 | *610449 | MYO7A | *276903 | NDUFA13 | *609435 | NDUFS6 | *603848 | NKX6-2 | *605955 |
| MMADHC | *611935 | MTERF4 | *615393 | MYO9A | *604875 | NDUFA2 | *602137 | NDUFS7 | *601825 | NLGN4X | *300427 |
| MME | *120520 | MTFMT | *611766 | MYOT | *604103 | NDUFA3 | *603832 | NDUFS8 | *602141 | NONO | *300084 |
| MMUT | *609058 | MTHFD1L | *611427 | MYPN | *608517 | NDUFA4 | *603833 | NDUFV1 | *161015 | NOTCH3 | *600276 |
| MOCOS | *613274 | MTHFR | *607093 | MYT1L | *613084 | NDUFA5 | *601677 | NDUFV2 | *600532 | NPC1 | *607623 |
| MOCS1 | *603707 | MTM1 | *300415 | NAA15 | *608000 | NDUFA9 | *603834 | NDUFV3 | *602184 | NPC2 | *601015 |
| MOCS2 | *603708 | MTMR14 | *611089 | NACC1 | *610672 | NDUFAF1 | *606934 | NEB | *161650 | NPHP1 | *607100 |
| MOGS | *601336 | MTMR2 | *603557 | NADK2 | *615787 | NDUFAF2 | *609653 | NECAP1 | *611623 | NPHP3 | *608002 |
| MORC2 | *616661 | MTO1 | *614667 | NAGA | *104170 | NDUFAF3 | *612911 | NEDD4L | *606384 | NPHP4 | *607215 |
| MPDU1 | *604041 | MTOR | *601231 | NAGLU | *609701 | NDUFAF4 | *611776 | NEFH | *162230 | NPL | *611412 |
| MPDZ | *603785 | MTPAP | *613669 | NAGS | *608300 | NDUFAF5 | *612360 | NEFL | *162280 | NPRL2 | *607072 |
| MPI | *154550 | MTR | *156570 | NALCN | *611549 | NDUFAF6 | *612392 | NEK1 | *604588 | NPRL3 | *600928 |
| MPV17 | *137960 | MTRR | *602568 | NANS | *605202 | NDUFB1 | *603837 | NEK8 | *609799 | NRAS | *164790 |
| MPZ | *159440 | MUSK | *601296 | NAPB | *611270 | NDUFB10 | *603843 | NEK9 | *609798 | NRXN1 | *600565 |
| MR1 | *600764 | MVK | *251170 | NARS2 | *612803 | NDUFB11 | *300403 | NEU1 | *608272 | NSDHL | *300275 |
| MRE11 | *600814 | MYBPC1 | *160794 | NAT8L | *610647 | NDUFB2 | *603838 | NEUROD1 | *601724 | NSUN2 | *610916 |
| MRPL12 | *602375 | MYCN | *164840 | NCAPD2 | *615638 | NDUFB3 | *603839 | NEUROG3 | *604882 | NT5C2 | *600417 |
| MRPL3 | *607118 | MYF6 | *159991 | NCAPD3 | *609276 | NDUFB4 | *603840 | NEXMIF | *300524 | NT5C3A | *606224 |
| NTNG1 | *608818 | PAH | *612349 | PDHX | *608769 | PGAM2 | *612931 | PIP5K1C | *606102 | POLG | *174763 |
| NTRK2 | *600456 | PAK1 | *602590 | PDK3 | *300906 | PGAP1 | *611655 | PKLR | *609712 | POLG2 | *604983 |
| NUAK1 | *608130 | PAK3 | *300142 | PDP1 | *605993 | PGAP2 | *615187 | PLA2G6 | *603604 | POLR1A | *616404 |
| NUBPL | *613621 | PANK2 | *606157 | PDP2 | *615499 | PGK1 | *311800 | PLAA | *603873 | POLR1C | *610060 |
| NUS1 | *610463 | PARK2 | *602544 | PDSS1 | *607429 | PGM1 | *171900 | PLCB1 | *607120 | POLR1D | *613715 |
| OAT | *613349 | PARK7 | *602533 | PDSS2 | *610564 | PHC1 | *602978 | PLD3 | *615698 | POLR2M | *606485 |
| OCLN | *602876 | PARS2 | *612036 | PDX1 | *600733 | PHF6 | *300414 | PLEC | *601282 | POLR3A | *614258 |
| OFD1 | *300170 | PAX3 | *606597 | PDYN | *131340 | PHF8 | *300560 | PLEKHG2 | *611893 | POLR3B | *614366 |
| OGDH | *613022 | PAX4 | *167413 | PDZD7 | *612971 | PHIP | *612870 | PLEKHG5 | *611101 | POLRMT | *601778 |
| OGT | *300255 | PAX5 | *167414 | PEPD | *613230 | PHKA1 | *311870 | PLOD1 | *153454 | POMGNT1 | *606822 |
| ONECUT1 | *604164 | PBX1 | *176310 | PET100 | *614770 | PHKA2 | *300798 | PLOD2 | *601865 | POMGNT2 | *614828 |
| OPA1 | *605290 | PC | *608786 | PET117 | *614771 | PHKB | *172490 | PLOD3 | *603066 | POMK | *615247 |
| OPA3 | *606580 | PCBD1 | *126090 | PEX1 | *602136 | PHKG2 | *172471 | PLP1 | *300401 | POMT1 | *607423 |
| OPHN1 | *300127 | PCCA | *232000 | PEX10 | *602859 | PHYH | *602026 | PLPBP | *604436 | POMT2 | *607439 |
| ORAI1 | *610277 | PCCB | *232050 | PEX11B | *603867 | PIBF1 | *607532 | PMM2 | *601785 | POU3F4 | *300039 |
| OSBPL2 | *606731 | PCDH12 | *605622 | PEX12 | *601758 | PIEZO2 | *613629 | PMP22 | *601097 | POU4F3 | *602460 |
| OTC | *300461 | PCDH15 | *605514 | PEX13 | *601789 | PIGA | *311770 | PMPCA | *613036 | PPIB | *123841 |
| OTOF | *603681 | PCDH19 | *300460 | PEX14 | *601791 | PIGC | *601730 | PMPCB | *603131 | PPM1D | *605100 |
| OTUD6B | *612021 | PCDH7 | *602988 | PEX16 | *603360 | PIGG | *616918 | PNKP | *605610 | PPOX | *600923 |
| OTX2 | *600037 | PCK1 | *614168 | PEX19 | *600279 | PIGH | *600154 | PNP | *164050 | PPP1CB | *600590 |
| OXCT1 | *601424 | PCK2 | *614095 | PEX2 | *170993 | PIGN | *606097 | PNPLA2 | *609059 | PPP1R15B | *613257 |
| P2RX2 | *600844 | PCNA | *176740 | PEX26 | *608666 | PIGO | *614730 | PNPLA4 | *300102 | PPP2R1A | *605983 |
| P3H1 | *610339 | PDE10A | *610652 | PEX3 | *603164 | PIGQ | *605754 | PNPLA6 | *603197 | PPP2R5D | *601646 |
| P4HB | *176790 | PDE6D | *602676 | PEX5 | *600414 | PIGS | *610271 | PNPLA8 | *612123 | PPP3CA | *114105 |
| PABPN1 | *602279 | PDGFB | *190040 | PEX6 | *601498 | PIK3CA | *171834 | PNPO | *603287 | PPT1 | *600722 |
| PACS1 | *607492 | PDHA1 | *300502 | PEX7 | *601757 | PIK3R2 | *603157 | PNPT1 | *610316 | PQBP1 | *300463 |
| PACS2 | *610423 | PDHA2 | *179061 | PFKFB2 | *171835 | PIK3R5 | *611317 | POGLUT1 | *615618 | PRDM8 | *616639 |
| PAFAH1B1 | *601545 | PDHB | *179060 | PFKM | *610681 | PINK1 | *608309 | POGZ | *614787 | PRICKLE1 | *608500 |
| PRICKLE2 | *608501 | PYROXD1 | *617220 | REEP1 | *609139 | RTN2 | *603183 | SCO2 | *604272 | SGCE | *604149 |
| PRKAG2 | *602743 | QARS1 | *603727 | REEP2 | *609347 | RTN4IP1 | *610502 | SCP2 | *184755 | SGCG | *608896 |
| PRKCG | *176980 | QDPR | *612676 | RELN | *600514 | RTN4R | *605566 | SCYL1 | *607982 | SGSH | *605270 |
| PRKRA | *603424 | QRSL1 | *617209 | REPS1 | *614825 | RTTN | *610436 | SDCCAG8 | *613524 | SH3TC2 | *608206 |
| PRNP | *176640 | RAB11B | *604198 | RERE | *605226 | RUBCN | *613516 | SDHA | *600857 | SHANK2 | *603290 |
| PRODH | *606810 | RAB18 | *602207 | RFT1 | *611908 | RXYLT1 | *605862 | SDHAF1 | *612848 | SHANK3 | *606230 |
| PRPF39 | *614907 | RAB23 | *606144 | RHOBTB2 | *607352 | RYR1 | *180901 | SDHAF2 | *613019 | SHH | *600725 |
| PRPS1 | *311850 | RAB33B | *605950 | RIT1 | *609591 | RYR3 | *180903 | SDHB | *185470 | SHOC2 | *602775 |
| PRRT2 | *614386 | RAB39B | *300774 | RLIM | *300379 | SACS | *604490 | SDHC | *602413 | SHROOM4 | *300579 |
| PRSS12 | *606709 | RAB3GAP1 | *602536 | RMND1 | *614917 | SAMD9L | *611170 | SELENOI | *607915 | SIGMAR1 | *601978 |
| PRUNE1 | *617413 | RAB3GAP2 | *609275 | RNASEH1 | *604123 | SAMHD1 | *606754 | SELENON | *606210 | SIK1 | *605705 |
| PRX | *605725 | RAB40AL | *300405 | RNASEH2A | *606034 | SARS1 | *607529 | SEMA3E | *608166 | SIL1 | *608005 |
| PSAP | *176801 | RAB7A | *602298 | RNASEH2B | *610326 | SARS2 | *612804 | SEPSECS | *613009 | SIN3A | *607776 |
| PSMD12 | *604450 | RAC1 | *602048 | RNASEH2C | *610330 | SASS6 | *609321 | SERAC1 | *614725 | SIX1 | *601205 |
| PTCD1 | *614774 | RAF1 | *164760 | RNASET2 | *612944 | SBF1 | *603560 | SERPINB6 | *173321 | SIX3 | *603714 |
| PTCH1 | *601309 | RALGAPA1 | *608884 | RNF170 | *614649 | SBF2 | *607697 | SERPINF1 | *172860 | SIX5 | *600963 |
| PTCHD1 | *300828 | RANBP2 | *601181 | RNF216 | *609948 | SC5D | *602286 | SERPINH1 | *600943 | SKI | *164780 |
| PTEN | *601728 | RAPSN | *601592 | ROBO1 | *602430 | SCARB2 | *602257 | SERPINI1 | *602445 | SLC12A5 | *606726 |
| PTPN11 | *176876 | RARS1 | *107820 | ROGDI | *614574 | SCN10A | *604427 | SET | *600960 | SLC12A6 | *604878 |
| PTPRQ | *603317 | RARS2 | *611524 | RORA | *600825 | SCN1A | *182389 | SETBP1 | *611060 | SLC13A5 | *608305 |
| PTS | *612719 | RBBP8 | *604124 | RORB | *601972 | SCN1B | *600235 | SETD2 | *612778 | SLC16A1 | *600682 |
| PUM1 | *607204 | RBCK1 | *610924 | RP2 | *300757 | SCN2A | *182390 | SETD5 | *615743 | SLC16A2 | *300095 |
| PURA | *600473 | RBFOX1 | *605104 | RPGRIP1L | *610937 | SCN3A | *182391 | SETDB2 | *607865 | SLC17A5 | *604322 |
| PUS1 | *608109 | RBFOX3 | *616999 | RPIA | *180430 | SCN4A | *603967 | SETX | *608465 | SLC17A8 | *607557 |
| PUS3 | *616283 | RBM12 | *607179 | RPL10 | *312173 | SCN7A | *182392 | SFXN4 | *615564 | SLC18A2 | *193001 |
| PYCR2 | *616406 | RBMX | *300199 | RPS23 | *603683 | SCN8A | *600702 | SGCA | *600119 | SLC18A3 | *600336 |
| PYGL | *613741 | RDX | *179410 | RPS6KA3 | *300075 | SCN9A | *603415 | SGCB | *600900 | SLC19A3 | *606152 |
| PYGM | *608455 | RECQL4 | *603780 | RRM2B | *604712 | SCO1 | *603644 | SGCD | *601411 | SLC1A1 | *133550 |
| SLC1A2 | *600300 | SLC35C1 | *605881 | SMC1A | *300040 | SPTBN2 | *604985 | SUOX | *606887 | TBL1XR1 | *608628 |
| SLC1A3 | *600111 | SLC37A4 | *602671 | SMCHD1 | *614982 | SPTBN4 | *606214 | SURF1 | *185620 | TBR1 | *604616 |
| SLC1A4 | *600229 | SLC39A13 | *608735 | SMPD1 | *607608 | SQSTM1 | *601530 | SYN1 | *313440 | TCAP | *604488 |
| SLC22A5 | *603377 | SLC39A14 | *608736 | SMPX | *300226 | SRD5A3 | *611715 | SYN2 | *600755 | TCF12 | *600480 |
| SLC25A1 | *190315 | SLC39A8 | *608732 | SMS | *300105 | SRGAP2 | *606524 | SYNE1 | *608441 | TCF3 | *147141 |
| SLC25A12 | *603667 | SLC3A1 | *104614 | SNAI2 | *602150 | SRGAP2C | *614704 | SYNE2 | *608442 | TCF4 | *602272 |
| SLC25A13 | *603859 | SLC45A1 | *605763 | SNAP25 | *600322 | SRPX2 | *300642 | SYNE4 | *615535 | TCF7L2 | *602228 |
| SLC25A15 | *603861 | SLC46A1 | *611672 | SNAP29 | *604202 | SSR4 | *300090 | SYNGAP1 | *603384 | TCN2 | *613441 |
| SLC25A19 | *606521 | SLC52A1 | *607883 | SNORD118 | *616663 | ST3GAL3 | *606494 | SYNJ1 | *604297 | TCOF1 | *606847 |
| SLC25A20 | *613698 | SLC52A2 | *607882 | SNX14 | *616105 | ST3GAL5 | *604402 | SYP | *313475 | TCTN1 | *609863 |
| SLC25A22 | *609302 | SLC52A3 | *613350 | SOD1 | *147450 | STAC3 | *615521 | SYT14 | *610949 | TCTN2 | *613846 |
| SLC25A26 | *611037 | SLC5A1 | *182380 | SON | *182465 | STAG1 | *604358 | SYT2 | *600104 | TCTN3 | *613847 |
| SLC25A3 | *600370 | SLC5A7 | *608761 | SOS1 | *182530 | STIL | *181590 | SZT2 | *615463 | TDGF1 | +187395 |
| SLC25A32 | *610815 | SLC6A1 | *137165 | SOS2 | *601247 | STIM1 | *605921 | TACO1 | *612958 | TDP1 | *607198 |
| SLC25A4 | *103220 | SLC6A17 | *610299 | SOX10 | *602229 | STRADA | *608626 | TAF1 | *313650 | TDP2 | *605764 |
| SLC25A42 | *610823 | SLC6A19 | *608893 | SOX11 | *600898 | STRC | *606440 | TAF13 | *600774 | TECPR2 | *615000 |
| SLC25A46 | *610826 | SLC6A3 | *126455 | SOX2 | *184429 | STT3A | *601134 | TAF2 | *604912 | TECR | *610057 |
| SLC26A4 | *605646 | SLC6A5 | *604159 | SOX3 | *313430 | STT3B | *608605 | TANGO2 | *616830 | TECTA | *602574 |
| SLC26A5 | *604943 | SLC6A8 | *300036 | SP7 | *606633 | STUB1 | *607207 | TARDBP | *605078 | TELO2 | *611140 |
| SLC2A1 | *138140 | SLC6A9 | *601019 | SPART | *607111 | STX1B | *601485 | TARS2 | *612805 | TENM4 | *610084 |
| SLC2A10 | *606145 | SLC9A1 | *107310 | SPAST | *604277 | STXBP1 | *602926 | TAT | *613018 | TERF2 | *602027 |
| SLC2A2 | *138160 | SLC9A6 | *300231 | SPATA5 | *613940 | STYXL1 | *616695 | TAZ | *300394 | TFAM | *600438 |
| SLC30A10 | *611146 | SLC9A9 | *608396 | SPEG | *615950 | SUCLA2 | *603921 | TBC1D23 | *617687 | TFG | *602498 |
| SLC30A9 | *604604 | SLITRK6 | *609681 | SPG11 | *610844 | SUCLG1 | *611224 | TBC1D24 | *613577 | TGFB1 | *190180 |
| SLC33A1 | *603690 | SMARCA1 | *300012 | SPG21 | *608181 | SUCO | *607723 | TBC1D7 | *612655 | TGFB2 | *190220 |
| SLC35A1 | *605634 | SMARCA4 | *603254 | SPG7 | *602783 | SUFU | *607035 | TBCD | *604649 | TGFB3 | *190230 |
| SLC35A2 | *314375 | SMARCB1 | *601607 | SPR | *182125 | SUGCT | *609187 | TBCE | *604934 | TGFBR1 | *190181 |
| SLC35A3 | *605632 | SMARCC2 | *601734 | SPTAN1 | *182810 | SUMF1 | *607939 | TBCK | *616899 | TGIF1 | *602630 |
| TGM6 | *613900 | TMEM70 | *612418 | TRIM54 | *606474 | TTN | *188840 | UCP2 | *601693 | VAC14 | *604632 |
| TH | *191290 | TMLHE | *300777 | TRIM63 | *606131 | TTPA | *600415 | UFC1 | *610554 | VAMP1 | *185880 |
| THAP1 | *609520 | TMTC3 | *617218 | TRIM8 | *606125 | TTR | *176300 | UFM1 | *610553 | VARS1 | *192150 |
| THOC2 | *300395 | TNIK | *610005 | TRIO | *601893 | TUBA1A | *602529 | UMOD | *191845 | VARS2 | *612802 |
| TIA1 | *603518 | TNNI2 | *191043 | TRIP12 | *604506 | TUBA8 | *605742 | UMPS | *613891 | VCP | *601023 |
| TIMM50 | *607381 | TNNT1 | *191041 | TRIP4 | *604501 | TUBB | *191130 | UNC13A | *609894 | VDAC1 | *604492 |
| TIMM8A | *300356 | TNNT3 | *600692 | TRIT1 | *617840 | TUBB2A | *615101 | UNC80 | *612636 | VDAC2 | *193245 |
| TJP2 | *607709 | TNPO3 | *610032 | TRMT10A | *616013 | TUBB2B | *612850 | UPB1 | *606673 | VLDLR | *192977 |
| TK2 | *188250 | TNXB | *600985 | TRMT10C | *615423 | TUBB3 | *602661 | UPF3B | *300298 | VMA21 | *300913 |
| TKT | *606781 | TOP3A | *601243 | TRMT5 | *611023 | TUBB4A | *602662 | UQCC3 | *616097 | VPS11 | *608549 |
| TLK2 | *608439 | TOR1A | *605204 | TRMU | *610230 | TUBB6 | *615103 | UQCR10 | *610843 | VPS13A | *605978 |
| TM4SF20 | *615404 | TOR1AIP1 | *614512 | TRNT1 | *612907 | TUBG1 | *191135 | UQCR11 | *609711 | VPS13D | *608877 |
| TMC1 | *606706 | TOR1AIP2 | *614513 | TROVE2 | *600063 | TUBGCP4 | *609610 | UQCRB | *191330 | VPS33A | *610034 |
| TMEM106B | *613413 | TPK1 | *606370 | TRPC3 | *602345 | TUBGCP6 | *610053 | UQCRC1 | *191328 | VPS37A | *609927 |
| TMEM107 | *616183 | TPM2 | *190990 | TRPV4 | *605427 | TUFM | *602389 | UQCRC2 | *191329 | VPS53 | *615850 |
| TMEM126A | *612988 | TPM3 | *191030 | TSC1 | *605284 | TUSC3 | *601385 | UQCRFS1 | *191327 | VRK1 | *602168 |
| TMEM126B | *615533 | TPP1 | *607998 | TSC2 | *191092 | TWIST1 | *601622 | UQCRH | *613844 | VWA3B | *614884 |
| TMEM132E | *616178 | TRAF3IP1 | *607380 | TSEN15 | *608756 | TWNK | *606075 | UQCRQ | *612080 | WAC | *615049 |
| TMEM138 | *614459 | TRAIP | *605958 | TSEN2 | *608753 | TXN2 | *609063 | UROC1 | *613012 | WARS1 | *191050 |
| TMEM165 | *614726 | TRAK1 | *608112 | TSEN34 | *608754 | TYMP | *131222 | UROD | *613521 | WARS2 | *604733 |
| TMEM199 | *616815 | TRAPPC11 | *614138 | TSEN54 | *608755 | UBA1 | *314370 | UROS | *606938 | WASHC4 | *615748 |
| TMEM216 | *613277 | TRAPPC12 | *614139 | TSFM | *604723 | UBA5 | *610552 | USH1C | *605242 | WASHC5 | *610657 |
| TMEM231 | *614949 | TRAPPC6B | *610397 | TSPAN7 | *300096 | UBE2A | *312180 | USH1G | *607696 | WDFY3 | *617485 |
| TMEM237 | *614423 | TRAPPC9 | *611966 | TTBK2 | *611695 | UBE3A | *601623 | USH2A | *608400 | WDPCP | *613580 |
| TMEM240 | *616101 | TREX1 | *606609 | TTC19 | *613814 | UBR3 | *613831 | USP18 | *607057 | WDR19 | *608151 |
| TMEM38B | *611236 | TRIM2 | *614141 | TTC21B | *612014 | UBR4 | *609890 | USP27X | *300975 | WDR26 | *617424 |
| TMEM43 | *612048 | TRIM32 | *602290 | TTC8 | *608132 | UBTF | *600673 | USP8 | *603158 | WDR33 | *618082 |
| TMEM67 | *609884 | TRIM36 | *609317 | TTI2 | *614426 | UCHL1 | *191342 | USP9X | *300072 | WDR45 | *300526 |
| WDR45B | *609226 | ZMYM3 | *300061 |  |  |  |  |  |  |  |  |
| WDR48 | *612167 | ZMYND11 | *608668 |  |  |  |  |  |  |  |  |
| WDR62 | *613583 | ZNF148 | *601897 |  |  |  |  |  |  |  |  |
| WDR73 | *616144 | ZNF335 | *610827 |  |  |  |  |  |  |  |  |
| WDR81 | *614218 | ZNF41 | *314995 |  |  |  |  |  |  |  |  |
| WFS1 | *606201 | ZNF423 | *604557 |  |  |  |  |  |  |  |  |
| WNT5A | *164975 | ZNF469 | *612078 |  |  |  |  |  |  |  |  |
| WWOX | *605131 | ZNF592 | *613624 |  |  |  |  |  |  |  |  |
| XDH | *607633 | ZNF674 | *300573 |  |  |  |  |  |  |  |  |
| XRCC1 | *194360 | ZNF711 | *314990 |  |  |  |  |  |  |  |  |
| XRCC4 | *194363 | ZNF81 | *314998 |  |  |  |  |  |  |  |  |
| YARS1 | *603623 | ZSWIM6 | *615951 |  |  |  |  |  |  |  |  |
| YARS2 | *610957 |  |  |  |  |  |  |  |  |  |  |
| YME1L1 | *607472 |  |  |  |  |  |  |  |  |  |  |
| YWHAG | *605356 |  |  |  |  |  |  |  |  |  |  |
| YY1 | *600013 |  |  |  |  |  |  |  |  |  |  |
| ZAK | *609479 |  |  |  |  |  |  |  |  |  |  |
| ZBTB18 | *608433 |  |  |  |  |  |  |  |  |  |  |
| ZBTB42 | *613915 |  |  |  |  |  |  |  |  |  |  |
| ZC3H14 | *613279 |  |  |  |  |  |  |  |  |  |  |
| ZDHHC15 | *300576 |  |  |  |  |  |  |  |  |  |  |
| ZDHHC9 | *300646 |  |  |  |  |  |  |  |  |  |  |
| ZEB2 | *605802 |  |  |  |  |  |  |  |  |  |  |
| ZFR | *615635 |  |  |  |  |  |  |  |  |  |  |
| ZFYVE26 | *612012 |  |  |  |  |  |  |  |  |  |  |
| ZFYVE27 | *610243 |  |  |  |  |  |  |  |  |  |  |
| ZIC2 | *603073 |  |  |  |  |  |  |  |  |  |  |
| ZMYM2 | *602221 |  |  |  |  |  |  |  |  |  |  |
