# Supplementary material for: Rapid Phenotype-Driven Gene Sequencing with the NeoSeq Panel: A Diagnostic Tool for Critically Ill Newborns with Suspected Genetic Disease
Source: J Clin Med. 2020 Jul 23;9(8):2362. doi: 10.3390/jcm9082362 (PMC7464859; doi:10.3390/jcm9082362)
Supplement: Supplementary file 1 [file jcm-09-02362-s001.zip › Table S2 .doc]

**Table S2.** Patient demographic and clinical data.

| **Case** | **Age** | **Sex** | **Phenotype: HPO** | **TAT** |
| --- | --- | --- | --- | --- |
| 1 | 25 d | M | Intrauterine growth retardation  Neonatal respiratory distress  Congenital microcephaly  Unilateral cryptorchidism  Neonatal hypoglycemia  Chronic metabolic acidosis  Lactic acidosis | 7d |
| 2 | 1 m, 15 d | M | Methylmalonic aciduria  Abnormality of myocardium  Chronic metabolic acidosis  Lactic acidosis | 7d |
| 3 | 2 m | F | Failure to thrive  Weight loss  Irritability  Infantile axial hypotonia  Developmental regression  Abnormality of serine metabolism  Hyperglycinemia | 4d |
| 4 | 20 d | F | Seizures  Epileptic encephalopathy | 6d |
| 5 | 18 d | M | Seizures  Epileptic encephalopathy | 7d |
| 6 | 25 d | F | Seizures  Epileptic encephalopathy | 6d |
| 7 | 6 d | M | Neonatal respiratory distress  Seizures  Epileptic encephalopathy  Chronic metabolic acidosis | 7d |
| 8 | 10 d | M | Vomiting  Hyperammonemia  Hypoketotic hypoglycemia  Fetal pyelectasis  Thyroid-stimulating hormone excess | 8d |
| 9 | 1 m | M | Generalized neonatal hypotonia  Seizures  Lethargy  Cerebellar atrophy | 10d |
| 10 | 3 d | F | Premature birth  Respiratory insufficiency due to muscle weakness  Generalized hypotonia  Encephalopathy | 10d |
| 11 | 9 d | F | Leukoencephalopathy  Cataracts  Renal cysts  Digestive bleeding | 5d |
| 12 | 12 d | M | Joint contractures  Microretrognathia  Microtia  Micropenis  Hypotonia  Hearing loss | 6 d |
| 13 | 15 d | F | Hirschsprung disease  Congenital hearing loss  Opsoclonus | 7 d |
| 14 | 22 d | F | Preeclampsia  Neonatal respiratory distress  Hypoglycemia | 7 d |
| 15 | 29 d | F | Intrauterine growth retardation  Meconium ileus  Neonatal respiratory distress  Generalized neonatal hypotonia  Hip dislocation  10 pairs of ribs | 10 d |
| 16 | 4 d | M | Focal seizures | 9 d |
| 17 | 1 m, 15 d | M | Hepatomegaly  Hypoglycemia  Ascites | 7 d |
| 18 | 2 m | M | Generalized neonatal hypotonia  Muscle weakness | 8 d |
| 19 | 2 m | M | Low birth weight  Generalized neonatal hypotonia  Meconium ileus  Failure to thrive  Congenital hip dislocation | 6 d |
| 20 | 10 d | F | Low birth weight  Generalized neonatal hypotonia  Microretrognathia  Ankyloblepharon  Hypotelorism  Limb dystonia | 8 d |
| 21 | 9 d | F | Patent ductus arteriosus  Pulmonary hypertension  Pulmonary hypoplasia  Diaphragmatic paralysis  Encephalopathy  Bilateral talipes equinovarus | 7 d |
| 22 | 16 d | M | Intrauterine growth retardation  Generalized neonatal hypotonia  Bilateral cryptorchidism  EEG with focal epileptiform discharges  Cerebral venous thrombosis | 7 d |
| 23 | 7 d | M | Absent left radius  Aplasia of the first finger  Atrial septal defect  Congenital hearing loss | 8 d |
| 24 | 2 m | F | Low birth weight  Right congenital diaphragmatic hernia  Aberrant right subclavian artery  Pancreatic dysplasia  Bilateral chorioretinal atrophy  Aplasia/hypoplasia of the vertebrae  Bilateral clinodactyly  Pineal cyst | 7 d |
| 25 | 17 d | F | Neonatal respiratory distress  Redundant neck skin  Subependymal cysts  Short neck  Narrow palate | 8 d |
| 26 | 26 d | M | Bilateral clubfoot  Feeding difficulties  Gastrostomy tube feeding in infancy  Gastroesophageal reflux  Generalized congenital hypotonia | 7 d |
| 27 | 1 m | M | Severe intrauterine growth retardation | 8 d |
| 28 | 1 m ,15 d | F | Seizures  Epileptic encephalopathy | 6 d |
| 29 | 9 d | F | Generalized neonatal hypotonia  Encephalopathy  Increased CSF lactic acid  Corneal stromal edema  Liver failure | 8 d |
| 30 | 13 d | F | Diffuse hyperinsulinism  Hyperammonemia | 8 d |
| 31 | 21 d | M | Encephalopathy  Hypertrophic cardiomyopathy | 7 d |
| 32 | 1 m, 4 d | F | Seizures  Epileptic encephalopathy | 10 d |
| 33 | 2 m | M | Seizures  Epileptic encephalopathy | 11 d |

Abbreviations: CSF, cerebrospinal fluid; d, day; m, month; F, female; HPO, human phenotype ontology; M, male; TAT, diagnostic turnaround time.
